# Combination of 4-1BB and DAP10 promotes proliferation and persistence of NKG2D(bbz) CAR-T cells

**DOI:** 10.3389/fonc.2022.893124

**Published:** 2022-07-29

**Authors:** Cheng Wei, Kangfu Xia, Yucheng Xie, Sishi Ye, Yanghui Ding, Zairu Liu, Rong Zheng, Jing Long, Qinchuan Wei, Yumei Li, Dongxia Yang, Xiaojun Xu, Ai Zhao, Jimin Gao

**Affiliations:** ^1^ Key Laboratory of Laboratory Medicine, Ministry of Education, School of Laboratory Medicine and Life Sciences, Wenzhou Medical University, Wenzhou, China; ^2^ Eye Hospital, School of Ophthalmology and Optometry, School of Biomedical Engineering, Wenzhou Medical University, Wenzhou, China; ^3^ Shandong Xinchuang Biotech, Jinan, China; ^4^ Department of Hematology, The Seventh Affiliated Hospital of Sun Yat-Sen University, Shenzhen, China; ^5^ Department of Geriatric, Affiliated Hangzhou First People’s Hospital, Zhejiang University School of Medicine, Hangzhou, China; ^6^ Zhejiang Qixin Biotech, Wenzhou, China

**Keywords:** NKG2D, CAR-T cell, 4-1BB, DAP10, PI3K

## Abstract

Chimeric antigen receptor (CAR)-T cell therapy has been shown to have considerable therapeutic effects in hematological malignancies, and NKG2D(z) CAR-T cell therapy has been verified to be safe based on clinical trials. However, due to the poor persistence of NKG2D(z) CAR-T cells, their therapeutic effect is not obvious. Here, we constructed NKG2D(bbz) CAR-T cells that can simultaneously activate 4-1BB and DAP10 costimulatory signaling. They were found to be cytotoxic to the target cells *in vitro* and *in vivo*. They exhibited low differentiation, low exhaustion, and good proliferation. Importantly, the proportions of central memory T (Tcm) and stem cell-like memory T (Tscm) cell subsets were strikingly increased. After long-term incubation with the target cells, they displayed reduced exhaustion compared to NKG2D(z) CAR-T cells. Further, in the presence of the phosphoinositide 3-kinase (PI3K) inhibitor LY294002, they exhibited reduced exhaustion and apoptosis, upregulated Bcl2 expression, and an increased proportion of Tcm cell subsets. Finally, NKG2D(bbz) CAR-T cells had better antitumor effects *in vivo*. In summary, the results showed that NKG2D(bbz) CAR-T cells may be valuable for cellular immunotherapy of cancer.

## Introduction

Chimeric antigen receptor (CAR)-T cell therapy has achieved significant success and attracted much attention in the treatment of hematologic cancers (1, 2). Since the successful US Food and Drug Administration (FDA) approval of the CAR-T cell therapy tisagenlecleucel (Kymriah) in 2017, several other CAR-T cell products for various hematological malignancies have been approved (3). The therapy involves using gene editing to allow T cells to express CARs that are not restricted by histocompatibility complex (MHC) molecules and can directly recognize and lyse tumor cells (4). The first-generation CARs had only a CD3ζ signaling domain, along with an extracellular antigen recognition domain. Modular construction has allowed them to be modified to create second- and third-generation CARs that connect CD28, 4-1BB, or both signaling domains to CD3ζ, in order to attempt to simulate the costimulation (signal 2) provided by antigen-presenting cells during T cell receptor (TCR) recognition, which is required for the activation of all physiological T cells (5–7).

CAR-T cells usually only recognize a single target, so antigen escape can easily occur due to antigen loss by cancer cells (8, 9). In contrast, we use the activated receptor natural-killer group 2, member D (NKG2D), which can recognize eight ligands, as the extracellular antigen recognition domain. NKG2D is mainly expressed on the surface of NK and CD8^+^ T cells (10). NKG2D ligands include MICA, MICB and ULBP1-6, which are expressed on the surface of most tumor cells but almost absent from the surface of normal cells (11). NKG2D(z) CAR (composed of CD3ζ and full-length NKG2D) T cells have been shown to be cytotoxic to multiple myeloma, lymphoma, and ovarian cancer cells in mice (12–14). Furthermore, the safety of these cells has been demonstrated in phase I clinical trials; however, due to their limited expansion and persistence, the therapeutic effect of these low-dose CAR-T cells was poor (15, 16).

T cells expressing CAR with a CD28 or 4-1BB signaling domain can both exert effective cytotoxicity, but differ in effector function, clinical efficacy, and toxicity. These differences are thought to be caused by differences in signaling cascade activation, leading to different subsets and viability of CAR-T cells (17–20). 4-1BB significantly increases the proportion of the central memory T (Tcm) cell subset and enhances the persistence and viability of CAR-T cells *in vivo*. DAP10 functions as a costimulatory molecule downstream of NKG2D in CD8^+^ T cells, thus enhancing the response of effector T cells, increasing cytokine secretion, and causing T cells to differentiate into memory precursor cells (21). Therefore, the activation of DAP10 costimulatory signaling by CARs may enable CAR-T cells to overcome immunosuppression encountered in the microenvironment of solid tumors (22–25). To enhance the persistence of NKG2D(z) CAR-T cells, we constructed an NKG2D(bbz) CAR structure involving full-length NKG2D, 4-1BB, and CD3ζ. NKG2D interacts with DAP10 of T cells and thereby recruits p85 protein, acting as a costimulatory molecule similar to CD28. Thus, the NKG2D(bbz) CAR-T cells can simultaneously activate 4-1BB and DAP10 costimulatory signaling.

## Materials and methods

### Vector construction, lentiviral production, and cell lines

Lentiviral vectors encoding NKG2D(z) and NKG2D(bbz) CAR were constructed by use of plenti-EF1α-MCS. NKG2D(z) CAR is composed of CD3ζ (52-164aa) and full-length NKG2D (1-216aa). NKG2D(bbz) CAR is composed of CD3ζ (52-164aa), 4-1BB (214-255aa), and full-length NKG2D (1-216aa). The cDNA sequences encoding these two CARs were codon-optimized, synthesized by GENEVA (Suzhou, China), and then cloned into plenti-EF1α-MCS. NKG2D(z) and NKG2D(bbz) lentiviruses were packaged in 293T cells (ATCC, Manassas, VA, USA) by use of a third-generation lentivirus packaging system (pLP1, pLP2, and pMD2.G).

The human cell lines MMIS, IM9, K562 and U266 (ATCC) were maintained in RPMI 1640 medium with 10% fetal bovine serum (FBS). The cell lines A549, ABC1 and MDA-MB-231 (ATCC) were maintained in DMEM with 10% FBS.

### Preparation of CAR-T cells

Peripheral blood mononuclear cells (PBMCs) from healthy donors were prepared *via* Fillco (TBD) density gradient centrifugation. The PBMCs were washed in buffer 1 (phosphate-buffered saline [PBS]+2%FBS) twice, and then the T cells were isolated and activated by CD3/CD28 magnetic beads (11141D, Gibco) at a ratio of 1:1. The T cells (at a density of 1.2×10^6^/mL) were cultured in serum-free medium (Corning) supplemented with 5% AB serum (Sigma), 100 U/mL IL2, 10 ng/mL IL7, and 10 ng/mL IL15 (Proteintech). After activation by CD3/CD28 magnetic beads for 24 h, the T cells were transduced with NKG2D(bbz) or NKG2D(z) lentiviruses. Cell viability and transduction efficiency were assessed 5 days after transduction.

### Flow cytometry

A BD FACS AriaII and a BD Accuri™ (BD Biosciences) were used to acquire data. Analysis was performed with FlowJo software (FlowJo). CAR expression was determined by surface staining with either antibody against NKG2D (APC/PE, Biolegend) or mCherry (mCherry was co-expressed in NKG2D CAR-T cells to more accurately assess the percentages of these cells).

CAR-T cell phenotypes were assessed with monoclonal antibodies against the following molecules: CD4 (FITC, Biolegend), CD8 (PE, Biolegend), PD1 (APC, Biolegend), Tim3 (FITC, Biolegend), LAG3 (PE, Biolegend), CD27 (PECY7, BD), CD28 (PECY7, BD), CD25 (APC, Biolegend), CD127 (APC, Biolegend), CD62L (PE/PB, Biolegend), CD45RA (APC/PE, Biolegend), CCR7 (FITC, Biolegend), annexin V (APC, Biolegend), and 7-aminoactinomycin D (7AAD; ThermoFisher). The Tscm subset is defined as CD45RA^+^CD45RO^+^CD62L^+^ T cells (26). NKG2D ligands were detected with antibodies against MICA/B (PE, Biolegend), ULBP1 (PE, R&D), ULBP2/5/6 (PE, R&D), ULBP3 (PE, R&D), and ULBP4 (PE, R&D). To assess Bcl2 expression, a Cytofix/Cytoperm Kit (BD Biosciences) was used to fix and permeabilize the cells and then the cells were labeled with antibody against Bcl2 (APC, Biolegend).

### Proliferation of NKG2D CAR-T cells *in vitro*

CAR-T cell labeling by carboxyfluorescein succinimidyl ester (CFSE) was used to evaluate the proliferation of NKG2D(bbz) CAR-T cells incubated with their specific ligand (soluble MICA [sMICA]-Fc fusion protein) without IL2/7/15. The cells were labeled with 2–5 μM CFSE at 4°C for 10 min followed by adding 9 mL buffer 1 to quench the reaction. The cells were washed twice with buffer 1 and then cultured with sMICA-Fc to stimulate NKG2D CAR-T cell proliferation without IL2/7/15. After 72 h, CFSE dilution, as an indicator of NKG2D CAR-T cell proliferation, was assessed by flow cytometry.

### Cytotoxicity assays

A549, ABC1 and MDA-MB-231 cells were seeded in E-Plate 16 (Acea Biosciences, San Diego, CA) at 1×10^4^ cells/well, and then monitored in a normal incubator overnight by use of an xCELLigence impedance-based real-time cell analysis (RTCA) system (Acea Biosciences). When the cells were in the logarithmic growth phase the next day, half of the medium was removed and replaced with normal or balanced medium containing NKG2D CAR-T cells or mock-T cells at an effector:target (E:T) ratio of 3:1. The cells were continuously monitored for several hours with the RTCA system, and the impedance was plotted over time.

Bioluminescence luciferase assays were also performed to measure the cytotoxic activity of CAR-T cells against NKG2D ligand-expressing target cells (K562, U266, MMIS, A549). The target cells (1×10^4^) were co-cultured with NKG2D CAR-T cells at various E:T ratios for 8 h with the mock-T cells serving as the negative control.

To assess cytokine production, A549 cells were plated at 5×10^4^ cells/well in 48-well plates and cultured in the presence or absence of 5×10^4^ NKG2D CAR-T cells/well. After 12 and 24 h, the medium was obtained to assess IL2, IFN-γ, GM-CSF, and TNF-α secretion by use of ELISA kits (LIANKE).

### *In vivo* anticancer assays

Five-to-6-week-old female NOD SCID gamma (NSG) mice (GemPharmatech) were intraperitoneally injected with 5×10^6^ A549-Luc-green fluorescent protein (GFP) cells. These cancer cells in the mice were then detected by live bioluminescent imaging. Images were collected and analyzed with a Xenogen-IVIS Imaging System. When the mean fluorescence intensity (MFI) value was >10^9^, the mice were divided into three groups, which received NKG2D(z) CAR-T cells (8×10^6^/mouse), NKG2D(bbz) CAR-T cells (8×10^6^/mouse), or mock-T cells (ethics ID number: Xmsq2021-0075). The weight of the mice and MFI values were regularly monitored. The supernatant was aspirated after centrifugation of peripheral blood from the mice (collected *via* the submandibular vein) and the secretion of IL6, IL2, IFN-γ, and TNF-α in the peripheral blood was measured with a Th1/Th2/Th17 kit (560484, BD Pharmingen). The percentages of NKG2D(z) and NKG2D(bbz) CAR-T cells were assessed based on mCherry fluorescence after lysis of erythrocytes.

### Statistical analyses

All statistical analyses were performed using GraphPad Prism v7.0. The data are reported as mean ± SD (n≥3). Two-way analysis of variance (ANOVA) followed by Dunnett’s multiple comparisons test were used to compare more than two groups, and two-tailed paired t-tests were used to compare two groups. P<0.05 was considered to indicate a significant difference.

## Results

### NKG2D ligand is highly expressed on the surface of some solid and blood cancer cells

The NKG2D ligands consist of eight cell-associated glycoproteins that belong to the MIC and ULBP families. We used flow cytometry to detect the expression of NKG2D ligands on the surface of multiple myeloma, liver cancer, breast cancer, and lung cancer cell lines. The results showed that the expression levels of NKG2D ligands on the surface of different tumor cells varied. The lung cancer cell line A549 highly expressed MICA, MICB, ULBP1, and ULBP2/5/6. The breast cancer cell line MB543 did not express ULBP1, but ULBP3 was partially expressed. The multiple myeloma cell line IM9 highly expressed MICA, MICB, and ULBP4 (**Supplementary Figure 1A**). To further verify the NKG2D ligands expression in solid tumor tissues, we performed immunohistochemical analysis to assess the expression in the tumor tissues of patients with gliomas, lipomas, and lung cancer. The results showed that MICA/B were highly expressed in the tumor tissues (**Supplementary Figure 1B**).

### Development of NKG2D CAR involving full-length NKG2D, 4-1BB, and CD3ζ

**Figure 1:**
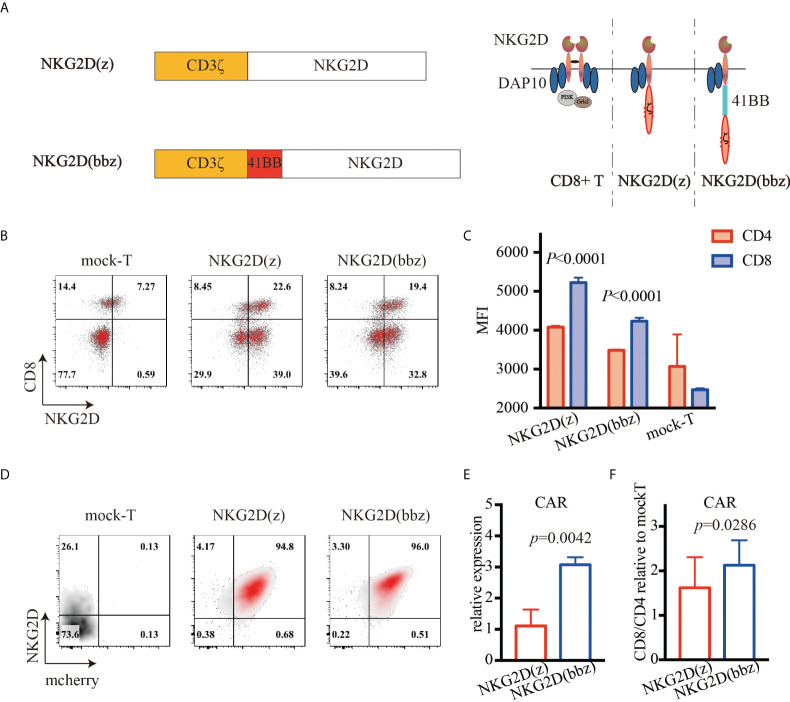
High expression of NKG2D(bbz) CAR in human T cells. **(A)** Graphical overview of NKG2D(bbz) CAR construct design. The CD3ζ signaling domain is followed by 4-1BB and full-length NKG2D. **(B)** 6 days after initial activation, NKG2D expression on the surface of CD4+ and CD8+ T cells was detected by flow cytometry. **(C)** Mean fluorescence intensity (MFI) of NKG2D on CD4+ and CD8+ NKG2D(z) and NKG2D(bbz) CAR-T cells. **(D)** NKG2D and mCherry expressed on T cells were detected by flow cytometry to assess the percentages of NKG2D(z) and NKG2D(bbz) CAR-T cells. **(E)** The numbers of NKG2D(z) and NKG2D(bbz) CAR-T cells were adjusted to be equivalent to each other. RNA was extracted to assess the expression of NKG2D CAR by qPCR. **(F)** CD4/CD8 ratio among NKG2D CAR-T cells was detected by flow cytometry. Data come from ≥3 donors. *p*<0.05 indicates significant difference. Data are presented as the mean ± SD.

NKG2D(z) CAR-T cells have been shown to be effective and safe in phase I clinical trials. However, due to their limited expansion and persistence, the therapeutic efficacy of low-dose CAR-T cells was poor (15, 16). Based on the results of clinical trials, we constructed NKG2D(bbz) CAR structures, involving full-length NKG2D, 4-1BB, and CD3ζ (**Figure 1A**). These cells could simultaneously activate DAP10 and 4-1BB costimulatory signaling. T cells were separated and extracted from the peripheral blood of healthy donors, activated with anti-CD3/CD28 magnetic beads for 24–48 h, and then transduced with NKG2D(z) or NKG2D(bbz) lentiviruses. On day 5, the expression of NKG2D CAR was detected on the surface of the T cells (**Figure 1B**). We found that the MFI of NKG2D on the surface of CD8^+^ NKG2D(bbz) CAR-T cells was 4232 ± 89 as opposed to 3485 ± 18.72 for CD4+ NKG2D(bbz) CAR-T cells (p<0.0001) (**Figure 1C**). To further compare and evaluate the differences in biological functions between NKG2D(z) and NKG2D(bbz) CAR-T cells, self-cleaving peptide 2A was used to co-express mCherry fluorescent protein based on both original CAR structures (**Supplementary Figure 2**; **Figures 1D, E**). We found that the ratio of CD8 to CD4 was 2.13 ± 0.56 for NKG2D(bbz) CAR-T cells and 1.62 ± 0.69 for NKG2D(z) CAR-T cells (*p*=0.0286) (**Figure 1F**).

### Cytotoxicity of NKG2D(bbz) CAR-T cells against the target cells

**Figure 2:**
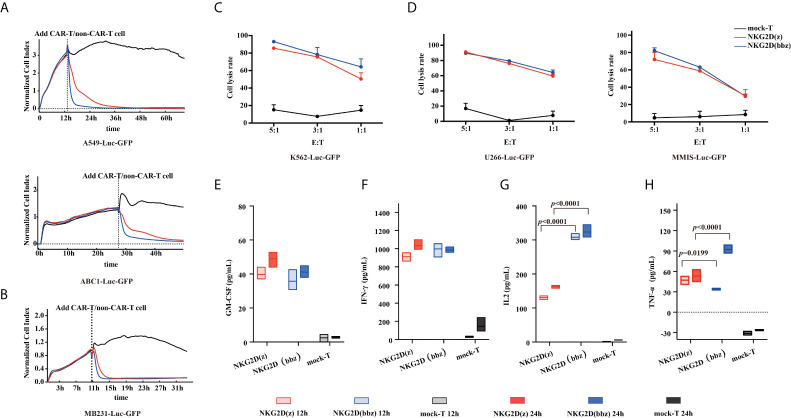
Characterization of NKG2D(bbz) CAR-T cell cytotoxicity and cytokine production *in vitro*. **(A, B)** Real-time cell analysis (RTCA) was used to monitor the cytolysis of A549, ABC1 and MDA-MB-231 cells by NKG2D(z) and NKG2D(bbz) CAR-T cells. Effector:target (E:T) cell ratio=1:1. Data are presented as mean ± SD of triplicate wells. **(C, D)** Cytotoxicity of NKG2D CAR-T cells was verified by co-incubation with luciferase-expressing U266, MMIS and K562 cells at the indicated E/T ratio. Data are presented as the mean ± SD of triplicate wells. **(E–H)** *In vitro* cytokine analysis of supernatants from co-culture of NKG2D(z) or NKG2D(bbz) CAR-T cells with A549 cells for 12 and 24 h. Data come from ≥3 donors. *p*<0.05 indicates significant difference. Data are presented as the mean ± SD.

To verify that NKG2D(bbz) CAR-T cells can recognize NKG2D ligands on tumor cells and have cytotoxic effects, A549, ABC1, MDA-MB-231, K562, MMIS and U266 cells were selected, and both the RTCA method (**Figures 2A, B**) and luciferase method (**Figures 2C, D**) were used. The NKG2D(bbz) CAR-T cells showed comparable cytotoxic activity to NKG2D(z) CAR-T cells *in vitro*.

We then used ELISA to compare cytokine secretion between the two CAR-T cells. After 12-h incubation with A549 target cells (E:T=1:1), the results showed that NKG2D(bbz) CAR-T cells secreted more IL2 compared to NKG2D(z) CAR-T cells (308 ± 9.404 vs 130.7 ± 5.334; *p*<0.0001) and NKG2D(z) CAR-T cells produced more TNF-α (47.17 ± 5.891 vs 33.85 ± 1.214; *p=*0.0199), while there was no significant difference in IFN-gamma; or GM-CSF secretion (**Figures 2E–H**).

### Superior proliferation of NKG2D(bbz) CAR-T cells

**Figure 3:**
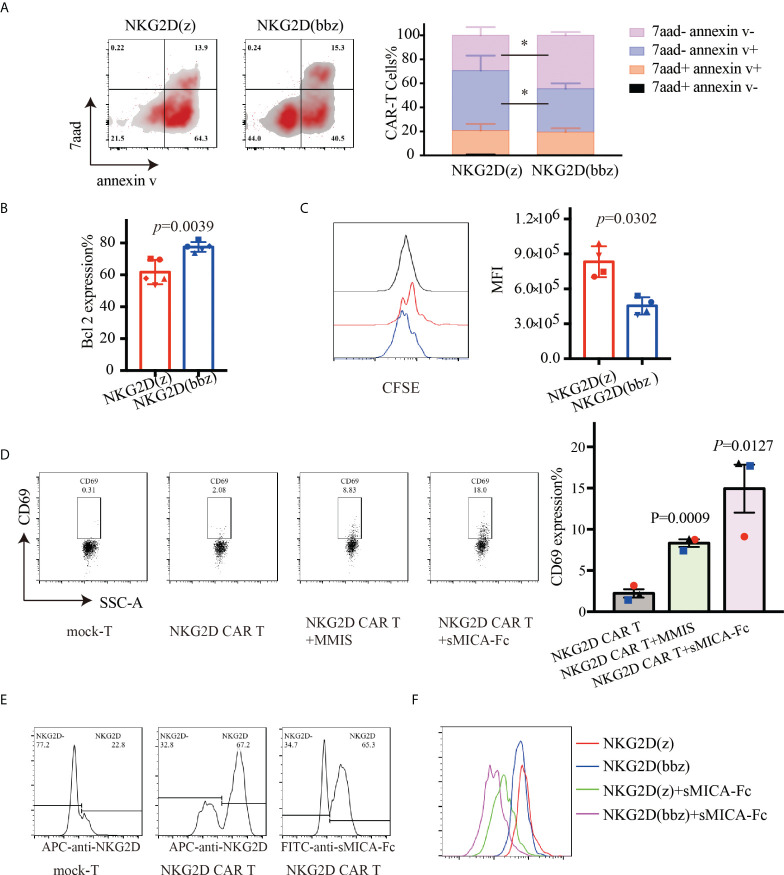
NKG2D(bbz) CAR-T cells (NKG2D(z) CAR-T cells modified with 4-1BB) exhibit decreased apoptosis and improved proliferation *in vitro*. The expression efficiency of NKG2D CAR on the surface of T cells was detected by flow cytometry 7 days after initial activation, and then the levels of NKG2D(z) and NKG2D(bbz) CAR-T cells were adjusted to be consistent with the level of mock-T cells and cultured in medium without IL2/7/15. **(A)** Apoptosis of NKG2D(z) and NKG2D(bbz) CAR-T cells was detected by flow cytometry 48 h later. The flow cytometry analysis and statistical analysis of apoptotic cells are presented from left to right. **(B)** Bcl2 expression in NKG2D(z) and NKG2D(bbz) CAR-T cells was detected by flow cytometry. **(C)** NKG2D(z) and NKG2D(bbz) CAR-T cells were labeled with carboxyfluorescein succinimidyl ester (CFSE), and their proliferation was assessed by flow cytometry after 3 days of culture. Data come from 3 donors. *p<0.05 indicates significant difference. Data are presented as the mean ± SD. **(D)** Soluble MICA (sMICA)-Fc protein was prepared using CHO cells and then co-incubated with NKG2D CAR-T cells for 24 h. The expression of CD69 on the surface of NKG2D CAR-T cells was detected using flow cytometry. MMIS target cells were co-incubated with NKG2D CAR-T cells as a positive control. **(E)** Flow cytometry was used to verify that sMICA-Fc can identify NKG2D on the surface of NKG2D CAR-T cells. NKG2D antibody was used as a positive control. **(F)** The specific ligand sMICA-Fc was used to stimulate the proliferation of CFSE-labeled NKG2D(z) and NKG2D(bbz) CAR-T cells, which was detected by flow cytometry.

To compare the persistence of NKG2D(bbz) and NKG2D(z) CAR-T cells in a nutrient-free environment, we measured the percentages and sizes of NKG2D CAR-T cells at day 7 after their initial activation (**Supplementary Figure 3**). After adjusting the NKG2D CAR expression levels to the equivalent extent, the culture conditions were changed so that there was no IL2/IL7/IL15/other cytokines. After 48 h of culture, the apoptosis of NKG2D CAR-T cells was detected by flow cytometry. We found that the percentage of viable cells was more significantly increased for NKG2D(bbz) CAR-T cells compared to NKG2D(z) CAR-T cells (44.5 ± 2.781 vs 29.4 ± 6.856; *p=*0.0233) (**Figure 3A**). Accordingly, we found that Bcl2 expression was also remarkedly upregulated in NKG2D(bbz) CAR-T cells compared to NKG2D(z) CAR-T cells (77.5 ± 3.07 vs 61.74 ± 7.647; *p=*0.0039) (**Figure 3B**). Moreover, we labeled the NKG2D CAR-T cells with CFSE and found that the MFI on the surface of NKG2D(bbz) CAR-T was 45396 ± 74387 as opposed to 833000 ± 132300 for NKG2D(z) CAR-T cells after 72 h of culture (*p=*0.0302) (**Figure 3C**). To further investigate the proliferation profiles of NKG2D(bbz) CAR-T cells in response to specific ligand stimulation, we co-incubated sMICA-Fc or MMIS myeloma cells (positive control) with NKG2D CAR-T cells for 24h. The results showed that 14.94 ± 5.048% of NKG2D CAR-T cells expressed CD69 after co-incubation with sMICA-Fc (*p=*0.0127) (**Figure 3D**). We also verified that NKG2D on the surface of NKG2D CAR-T cells could be recognized by sMICA-Fc by use of flow cytometry. The sMICA-Fc could also be used to detect the percentage of NKG2D CAR-T cells (**Figure 3E**). In addition, we found that NKG2D(bbz) CAR-T cells had faster proliferation in the presence of sMICA-Fc and absence of IL2/7/15 (**Figure 3F**).

### NKG2D(bbz) CAR-T cells resisted exhaustion and exhibited reduced differentiation *in vitro*

**Figure 4:**
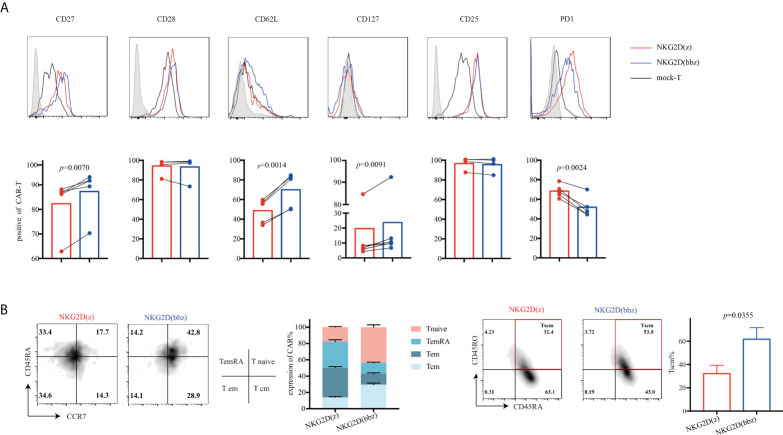
Compared to NKG2D(z) CAR-T cells, NKG2D(bbz) CAR-T cells exhibited reduced differentiation and exhaustion, and the Tnaive cell subset was increased. **(A)** CD27, CD28, CD127, CD25, CD62L, and PD1 on the surface of NKG2D(z) and NKG2D(bbz) CAR-T cells were detected by flow cytometry at 9 days after initial activation. The curves represent the flow cytometry peak map, and the bars represent the statistical map of the corresponding marker. **(B)** T cell subsets of NKG2D(z) and NKG2D(bbz) CAR-T cells were analyzed. T cell subsets are divided into naïve (CD45RA+, CCR7+), effector memory (em) (CD45RA−, CCR7−), central memory (cm) (CD45RA−, CCR7+), terminal effectors re-expressing CD45RA (emRA) (CD45RA+, CCR7−), and stem cell-like memory (scm) (CD45RA+, CD45RO+). Data come from ≥3 donors. *p*<0.05 indicates significant difference. Data are presented as the mean ± SD.

To analyze the phenotypic differences between NKG2D(z) and NKG2D(bbz) CAR-T cells, we examined the cells by flow cytometry on day 9 after initial activation. We found that NKG2D(z) CAR-T cells had lower CD27 (82.32 ± 10.82 vs 87.3 ± 9.545; *p=*0.0070), CD127 (19.64 ± 31.86 vs 23.72 ± 33.61; *p=*0.0091), and CD62L (48.64 ± 12.21 vs 69.98 ± 17.79; *p=*0.0014) expression and higher PD1 (68.44± 6.734 vs 51.88 ± 10.65; *p=*0.0024) expression, but NKG2D(bbz) CAR-T cells exhibited lower differentiation and exhaustion (**Figure 4A**). Additionally, the distribution of NKG2D CAR-T cell subsets was examined and NKG2D(bbz) CAR-T cells were found to have more Tnaive (CD45RA^+^, CCR7^+^) (43.73 ± 2.914 vs 17.67 ± 0.9504 for NKG2D(bbz) CAR-T cells and NKG2D(z) CAR-T cells, p<0.0001), Tcm (CD45RA^-^, CCR7^+^) (29.53 ± 1.93 vs 13.93 ± 0.9074, *p*<0.0001), and Tscm (CD45RO^+^, CD45RA^+^) (61.75 ± 9.882 vs 32.25 ± 7.709; *p=*0.0355) cell subsets (**Figure 4B**). Taken together, these findings demonstrated that the addition of the costimulatory 4-1BB to the NKG2D(z) CAR structure effectively reduced NKG2D CAR-T cell differentiation and exhaustion.

### NKG2D(bbz) CAR-T cells exhibited better antitumor effects *in vivo*

**Figure 5:**
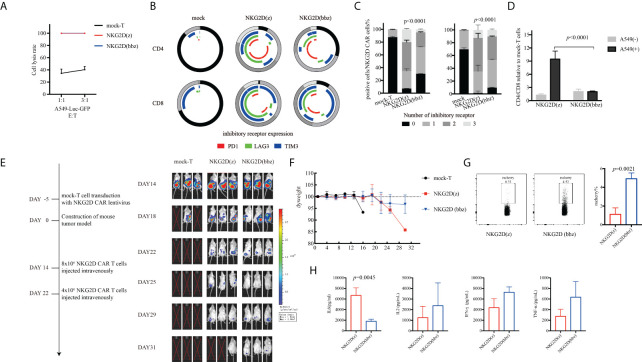
Compared to NKG2D(z) CAR-T cells, NKG2D(bbz) CAR-T cells co-cultured with A549 cells had lower exhaustion and could effectively inhibit tumor growth *in vivo*. **(A)** NKG2D(z) and NKG2D(bbz) CAR-T cells were incubated with A549-Luc-GFP cells for 96 h, and the luciferase method was used to evaluate their long-term tumor cell inhibition. **(B, C)** After NKG2D(z) and NKG2D(bbz) CAR-T cells were co-incubated with A549-Luc-GFP cells for 96 h, NKG2D(z) and NKG2D(bbz) CAR-T cells were collected, and PD1, Tim3, and LAG3 on the surface were assessed. Data come from ≥3 donors. *p*<0.05 indicates significant difference. **(D)** Analysis of CD4/CD8 ratio among NKG2D(z) and NKG2D(bbz) CAR-T cells by flow cytometry. Data come from ≥3 donors. *p*<0.05 indicates significant difference. **(E)** NOD SCID gamma (NSG) mice were intraperitoneally injected with 5×10^6^ A549-Luc-GFP cells. 14 days later, 8×10^6^ NKG2D CAR-T cells were intravenously injected. Tumor burden was monitored using bioluminescence intensity based on a Xenogen-IVIS imaging system. **(F)** Analysis of the weight of mice. Data are presented as the mean ± SD. **(G)** Percentage of NKG2D(z) and NKG2D(bbz) CAR-T cells in the peripheral blood of mice was detected by flow cytometry. **(H)** IL2, IL6, IFN-gamma;, and TNF-α levels in peripheral blood of mice were detected using a human Th1/Th2/Th17 cytometric bead array kit.

To further observe the antitumor ability of NKG2D(bbz) and NKG2D(z) CAR-T cells, we incubated them with A549 cells for 96 h and then assessed their cytotoxicity by use of luciferase assays. We found that there was no significant difference in cytotoxicity after long-term incubation with tumor target cells *in vitro* between NKG2D(bbz) and NKG2D(z) CAR-T cells (**Figure 5A**). In addition, we collected NKG2D CAR-T cells after co-incubation to assess the exhaustion of CD4+ or CD8+ NKG2D CAR-T cells and found that CD4^+^ and CD8^+^ NKG2D(z) CAR-T cells were exhausted earlier than corresponding NKG2D(bbz) CAR-T cells (**Figures 5B, C**). Moreover, the CD8 subsets among NKG2D(z) CAR-T cells were more likely to die after 4 days of incubation (**Figure 5D**). Based on these results, we constructed a A549-bearing mouse model and found that NKG2D(bbz) CAR-T-cell-treated mice exhibited lasting anti-tumor effects with two infusions on day 14 and day 22 (**Figures 5E, F**). However, there was no significant difference in the body weight of these NKG2D CAR-T-treated mice. On day 29, we assessed the levels of IL2, IL6, IFN-gamma;, and TNF-α in the peripheral blood of the mice and found that IL6 was significantly higher in the NKG2D(z) CAR-T-treated group than in the NKG2D(bbz) CAR-T-treated group (6689 ± 1414 vs 1794 ± 395.1; *p=*0.0045), while the percentage of NKG2D(bbz) CAR-T cells in peripheral blood was 4.917 ± 0.6191 compared to 1.11± 0.6942 for NKG2D(z) CAR-T cells (*p=*0.0021) (**Figures 5G, H**).

### Phosphoinositide 3-kinase (PI3K) inhibitor enhanced persistence of NKG2D CAR-T cells

**Figure 6:**
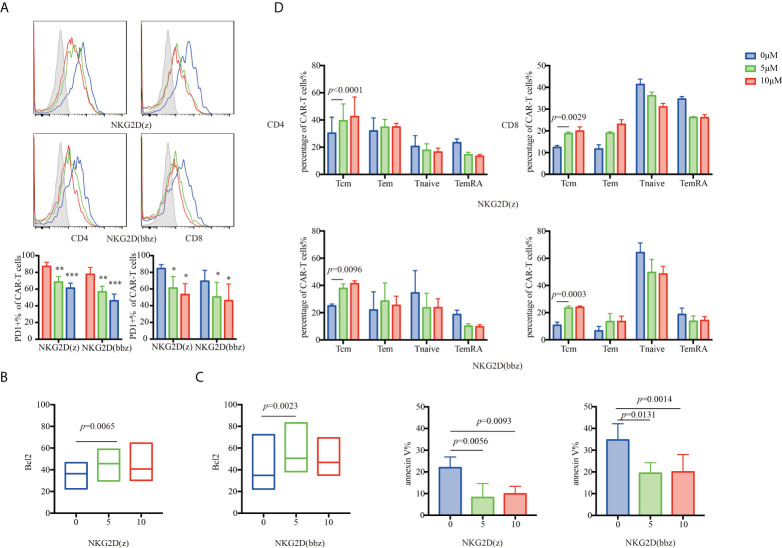
The appropriate concentration of a PI3K inhibitor (LY294002) can partially resist NKG2D(z) and NKG2D(bbz) CAR-T cell exhaustion caused by the PI3K pathway by upregulating Bcl2. **(A)** LY294002 (0, 5, or 10 µM) was added to NKG2D CAR-T cells. After 48 h of culture, PD1 on the surface of CD4+ and CD8+ NKG2D(z) and NKG2D(bbz) CAR-T cells was assessed. **(B, C)** Bcl2 and annexin V expressed by NKG2D CAR-T cells were assessed by flow cytometry. **(D)** After NKG2D(z) and NKG2D(bbz) CAR-T cells received LY294002 at different concentrations for 48 h, the subset classification of NKG2D CAR-T cells was assessed by flow cytometry. Data come from ≥3 donors. *p*<0.05 indicates significant difference. *p<0.05, **p<0.01, ***p<0.001. Data are presented as the mean ± SD.

NKG2D(z) CAR-T cells are prone to apoptosis during culture. The PI3K inhibitor LY294002 has been found to effectively improve the viability of these cells (27). Therefore, we investigated the effect of the PI3K inhibitor on NKG2D(bbz) CAR-T cells (**Figure 6**). At 7 days after initial activation of NKG2D(bbz) CAR-T cells, the PI3K inhibitor at various concentrations (0, 5, or 10 µM) was added and the cells were cultured for 48 h.

PD1 expression on the surface of CD4^+^/CD8^+^ NKG2D(bbz) CAR-T cells was significantly decreased after adding 5 μM of PI3K inhibitor (77.75 ± 8.15 vs 56.75 ± 6.695 for CD4+NKG2D(bbz) CAR-T cells treated with 0 and 5 µM, respectively, *p=*0.0013; 69.37 ± 12.99 vs 50.43 ± 17.49 for CD8+NKG2D(bbz) CAR-T cells treated with 0 and 5 µM, respectively, *p=*0.0184) (**Figure 6A**).

To explore how the PI3K inhibitor reduced NKG2D CAR-T cell exhaustion, we assessed Bcl2 and annexin V expression and found that the PI3K inhibitor slightly increased Bcl2 expression in both NKG2D(z) CAR-T cells (36.64± 10.11 vs 43.08 ± 11.93 for 0 and 5 µM, respectively, *p=*0.0065) and NKG2D(bbz) CAR-T cells (46.6 ± 22.99 vs 58.96 ± 22.55 for 0 and 5 µM, respectively, *p=*0.0023) (**Figures 6B, C**), and their Tcm cell subset significantly increased (24.96 ± 1.436 vs 37.8 ± 3.316 for 0 and 5 µM of CD4+NKG2D(bbz) CAR-T cells, *p=*0.0096; 10.68 ± 2.311 vs 23.4 ± 1.485 for 0 and 5 µM of CD8+NKG2D(bbz) CAR-T cells, *p=*0.0003) (**Figure 6D**). In summary, the results revealed that inhibition of PI3K signaling could effectively inhibit NKG2D CAR-T cell apoptosis, and thus a PI3K inhibitor may be used to improve their survival when NKG2D(bbz) CAR-T cells are cultured or used *in vivo*.

## Discussion

NKG2D, which is involved in innate and adaptive immunity, is a type II transmembrane activating receptor that is mainly expressed on CD8^+^ T cells and NK cells (28–30). The approval of CD19-directed CAR-T cell therapy for B-cell hematological malignancies provides strong clinical validation for CAR-T therapy, and thus provides an impetus for the development of CAR-T cell therapy for other cancers.

Several studies have verified the antitumor ability of NKG2D(z) CAR-T cells in mice with ovarian cancer and multiple myeloma (13, 31). The safety of NKG2D(z) CAR-T cells has also been demonstrated in clinical trials of myelodysplastic syndromes/acute myeloid leukemia and relapsed/refractory multiple myeloma (15, 16). However, these cells were found to have only short-term persistence *in vivo* during the treatment (32). In this study, we prepared NKG2D(bbz) CAR-T cells that could simultaneously activate 4-1BB and DAP10 costimulatory signaling. To minimize the interference of NKG2D expression on CD8^+^ T cells and better compare the differences between NKG2D(z) and NKG2D(bbz) CAR-T cells, we used self-cleaving peptide 2A to co-express mCherry fluorescent protein. NKG2D(bbz) CAR-T cells had more Tnaive and Tcm cell subsets with higher CD27 and CD62L expression *in vitro*. 4-1BB costimulatory signaling makes it easier for CAR-T cells to differentiate into Tcm cells, reduces cell exhaustion, and prolongs the life of CAR-T cells *in vivo* (33, 34). NKG2D interacts with DAP10, which contains a YINM motif and thereby recruits p85 to induce PI3K signaling and Grb2 to activate Vav-SOS signaling (35, 36). Similar to CD28, the NKG2D-DAP10 complex eventually leads to AP-1, NFAT, and NF-κB nuclear translocation and subsequent cell survival, proliferation, upregulated expression of effector molecules and cytokines, and the release of cell lysate particles after a series of cascade reactions (10, 37, 38).

Regarding the use of CAR-T cell therapy for solid tumors, the tumor microenvironment is inhospitable to immune cell proliferation (39). The signaling domains of the co-receptor CD28 and 4-1BB affect the metabolic characteristics of human CAR-T cells, including enhancing cell persistence in the tumor microenvironment (33, 40). Compared to NKG2D(z) CAR-T cells, NKG2D(bbz) CAR-T cells had faster proliferation and less apoptosis in the absence of IL2/7/15, while PD1 expression was lower under normal culture conditions. Meanwhile, we showed that NKG2D(bbz) CAR-T cells proliferate faster under the stimulation of their specific ligand sMICA-Fc. After NKG2D(z) and NKG2D(bbz) CAR-T cells were co-cultured with target tumor cells for 96 h, NKG2D(bbz) CAR-T cells exhibited lower exhaustion and secreted more IL2 within 24 h than NKG2D(z) CAR-T cells. Other studies have also shown that 4-1BB could prolong the persistence of CAR-T cells *in vivo* and ultimately enhance their antitumor ability (41, 42). In the current study, after the second infusion of NKG2D CAR-T cells, the mice in the NKG2D(z)-treated group showed significant weight loss and higher IL6, which may lead to inflammation and death of the mice. After prolonged incubation with A549 cells, the reduced proportion of CD8^+^ NKG2D(z) cells may be related to the fact that CD8^+^ NKG2D(z) CAR-T cells were more easily lost, indicating that CD4^+^ and CD8^+^ NKG2D CAR-T cells should be prepared separately in the future to study the differences in target tumor cell lysis and cytokine secretion.

Many studies have shown that PI3K inhibition can overcome target-driven “self-fratricide” of NKG2D CAR-T cells through reducing the expression of NKG2D CAR on the CAR-T cell surface (25). In our study, we found that inhibition of PI3K activation significantly increased the Tcm subset of NKG2D(bbz) CAR-T cells *in vitro*, suggesting that the treated NKG2D(bbz) CAR-T cells had improved capacities for long-term survival. We also found that inhibition of PI3K activation with LY294002 partially reduced the apoptosis of NKG2D(z) and NKG2D(bbz) CAR-T cells and up-regulated Bcl2 expression. PI3K could decrease the persistence and impair the function of CAR-T cells *in vivo* (43, 44). When CAR-T cells were cultured *in vitro*, the addition of IL15 could reduce mTORC1 activity so as to induce anti-apoptotic properties associated with up-regulated Bcl2 expression (45). Thus, IL15 and LY294002 together may further reduce mTORC1 activity to up-regulate Bcl2 expression. In the future, NKG2D(bbz) CAR-T cells and a PI3K inhibitor may be combined *in vitro* and *in vivo* to improve their persistence and anticancer effects.

In summary, we constructed NKG2D(bbz) CAR-T cells that could simultaneously activate both 4-1BB and DAP10 costimulatory signaling. To minimize the interference caused by the expression of natural NKG2D on the surface of CD8^+^ T cells, we made NKG2D(z) and NKG2D(bbz) CAR-T cells co-express mCherry fluorescent protein. Compared to NKG2D(z) CAR-T cells, NKG2D(bbz) CAR-T cells exhibited low differentiation and reduced exhaustion *in vitro*, and also sustained anticancer activity *in vivo*. Moreover, we found that a PI3K inhibitor reduced apoptosis and upregulated Bcl2 expression, and increased the proportion of the Tcm cell subset. Therefore, our study provides further experimental evidence for the clinical application of NKG2D(bbz) CAR-T cells, potentially with a PI3K inhibitor.

## Data availability statement

The original contributions presented in the study are included in the article/**supplementary material**. Further inquiries can be directed to the corresponding authors.

## Ethics statement

The animal study was reviewed and approved by the tab of animal experimental ethical inspection of laboratory animal center, Wenzhou Medical University.

## Author contributions

JG,AZ, XX and CW designed the study. CW interpreted the data and wrote the manuscript. CW, KX, YX, YD, SY, ZL, QW, YL, DY, JL, and RZ performed the experiments. All authors revised the manuscript and approved the final version of the manuscript.

## Funding

This study was partially funded by Construction Fund of Key Medical Disciplines of Hangzhou (No.OO20200055), Wenzhou Municipal Science and Technology Research Program (ZS2017014, 2018ZY001), Scientific Research Fund of the National Health Commission of PR China (WKJ-ZJ-1928), and Shandong Provincial Key R & D programs (2021CXGC011102), Sanming Project of Medicine in Shenzhen (No. SZSM201911004) and Shenzhen Science and Technology Plan Basic Research Project (No. JCYJ20180307150408596).

## Conflict of interest

Authors AZ and JG was were employed by Zhejiang Qixin Biotech.

The remaining authors declare that the research was conducted in the absence of any commercial or financial relationships that could be construed as a potential conflict of interest.

## Publisher’s note

All claims expressed in this article are solely those of the authors and do not necessarily represent those of their affiliated organizations, or those of the publisher, the editors and the reviewers. Any product that may be evaluated in this article, or claim that may be made by its manufacturer, is not guaranteed or endorsed by the publisher.
