# Supplementary material for: Combination of 4-1BB and DAP10 promotes proliferation and persistence of NKG2D(bbz) CAR-T cells
Source: Front Oncol. 2022 Jul 29;12:893124. doi: 10.3389/fonc.2022.893124 (PMC9372572; doi:10.3389/fonc.2022.893124)
Supplement: Supplementary file 1 [file DataSheet_1.docx]

Supplementary Material

Supplementary method

Immunohistochemistry

Tumor tissue sections of patients were taken for dewaxing and rehydration, antigen repair, closure using 20% goat serum, and incubated with anti-MICB MAb (R&D MAB13001) overnight at 4°C. Visual staining of Bound primary Ab was performed with Mouse / Rabbit Enhanced Polymer Detection System kit (ZSGB-BIO PV9000) according to the manufacturer's instructions. Final sections were stained with hematoxylin.

Supplementary Figures


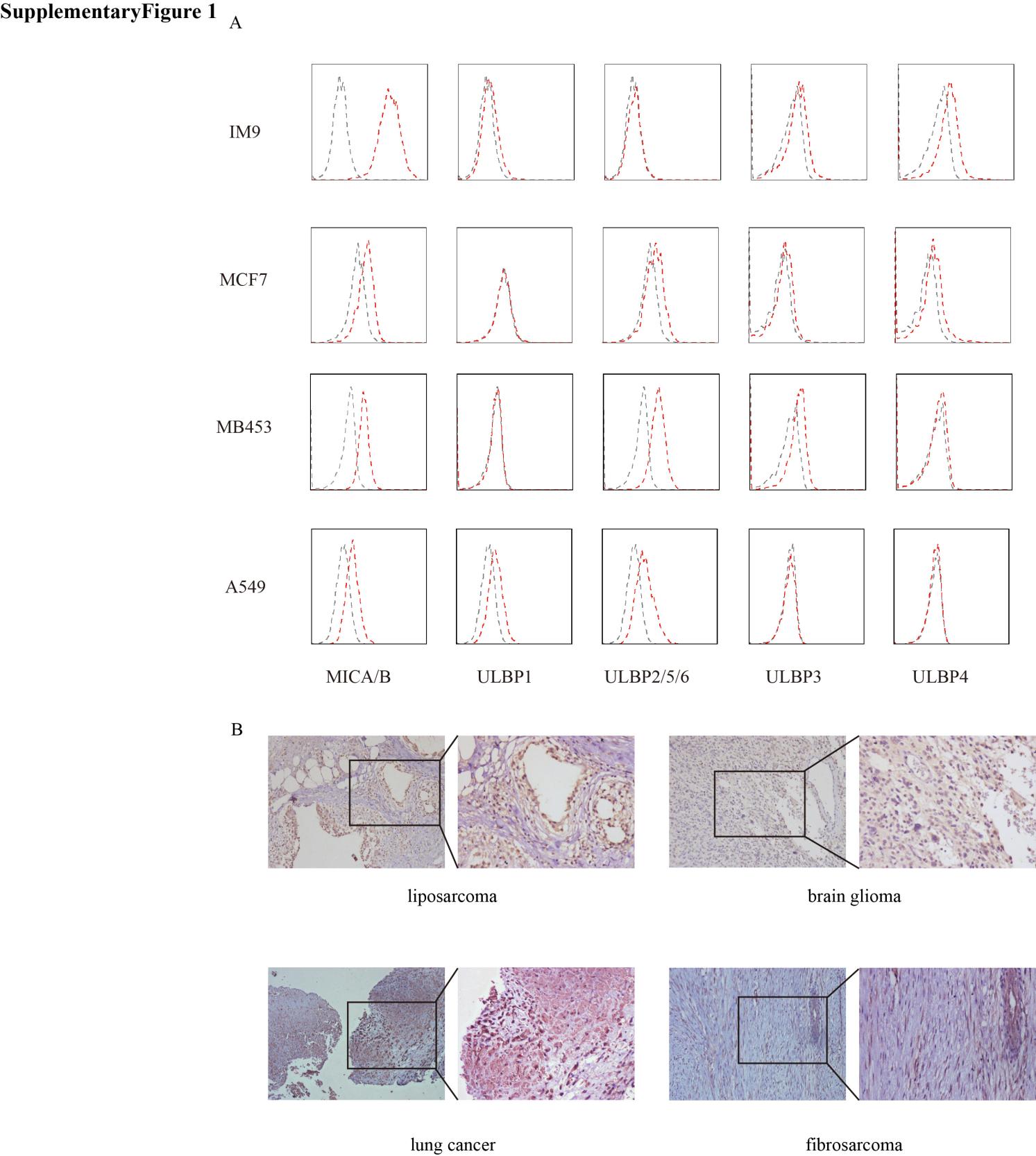


Supplementary Figure 1. NKG2D ligands are highly expressed on various tumor cell surfaces. (A) Flow cytometry was used to detect the eight NKG2D ligands on IM9, MCF7, MB453, and A549 cell surfaces. Three independent experiments were conducted, and the figure shows representative results. (B) Immunohistochemistry was used to detect the expression of MICA/B (NKG2D ligands) in glioma, liposarcoma, lung cancer, and fibrosarcoma.


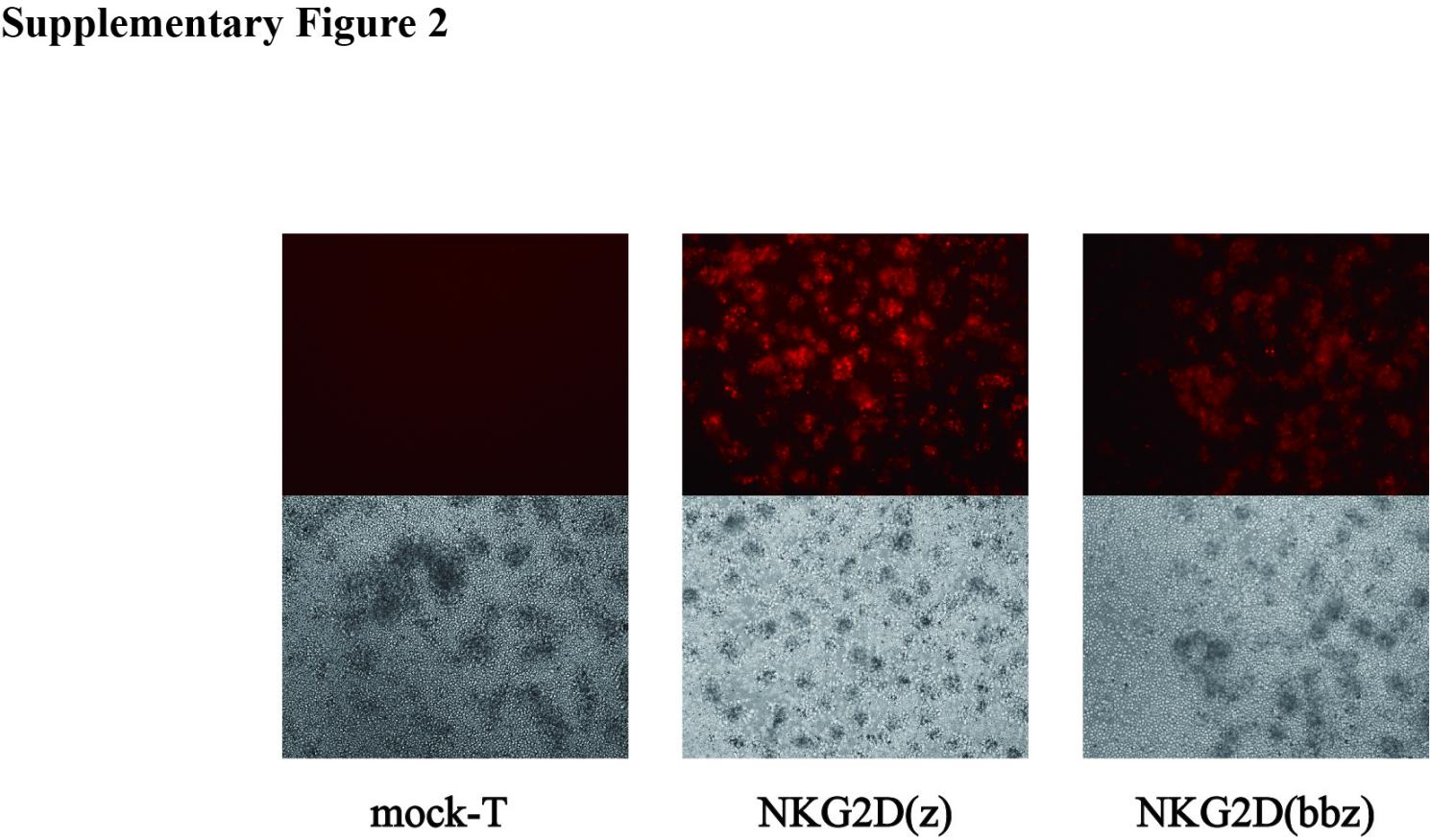


Supplementary Figure 2. The mcherry fluorescence can be observed by fluorescence microscopy on day 7 of NKG2D CAR T cell preparation..


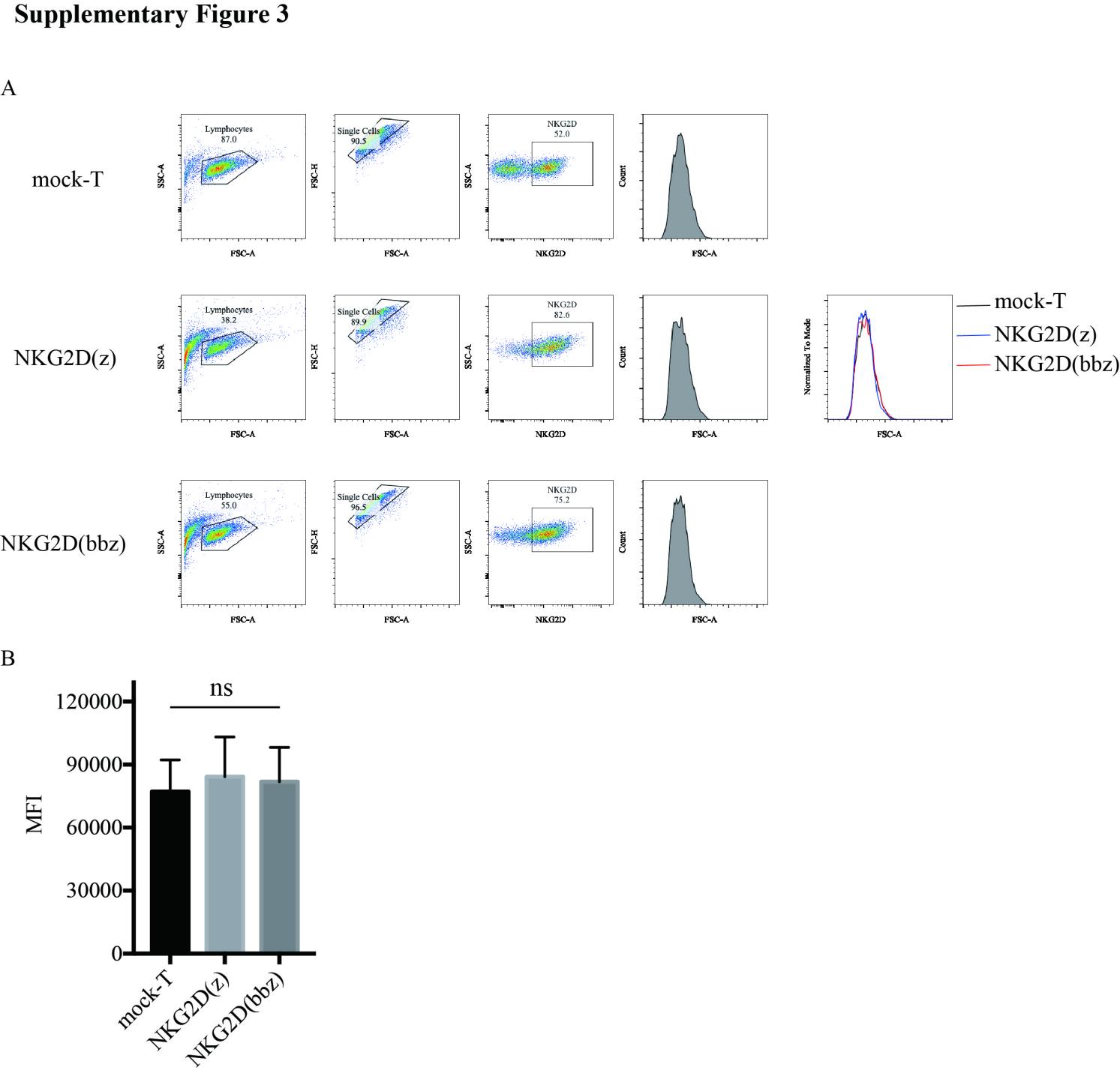


Supplementary Figure 3. There was no significant difference in the size of NKG2D(z) and NKG2D(bbz) CAR T cells and mock-T cells at day 7 of initial activation. (A,B) On day 5 of initial activation, anti-CD3/CD28 magnetic beads were removed using a magnetic holder, and after two days of culture, the size of NKG2D+ T cells was assessed using flow cytometry. (A) the flow cytometry circle gate method, (B) the statistical plot of NKG2D(z), NKG2D(bbz), and mock-T cells sizes.
